# Exploration of Hypoglycemic Activity of *Saccharomyces pastorianus* Extract and Evaluation of the Molecular Mechanisms

**DOI:** 10.3390/molecules26144232

**Published:** 2021-07-12

**Authors:** Chien-Hui Wu, Chung-Hsiung Huang, Ming-Chuan Chung, Shun-Hsien Chang, Guo-Jane Tsai

**Affiliations:** 1 Department of Seafood Science, National Kaohsiung University of Science and Technology, Nanzi 81157, Taiwan; chwu@nkust.edu.tw; 2 Department of Food Science, National Taiwan Ocean University, Keelung 20224, Taiwan; huangch@mail.ntou.edu.tw (C.-H.H.); s010135@hotmail.com (M.-C.C.); 3 Institute of Food Safety and Risk Management, National Taiwan Ocean University, Keelung 20224, Taiwan; lewis@mail.ntou.edu.tw; 4 Center for Marine Bioscience and Biotechnology, National Taiwan Ocean University, Keelung 20224, Taiwan

**Keywords:** diabetes, glucose-6-phosphate dehydrogenase, type 4 glucose transporter, hypoglycemic protein, *Saccharomyces pastorianus*

## Abstract

Although the hypoglycemic potential of brewer’s yeast extract has been reported, there is limited information pertaining to the hypoglycemic ingredients of *Saccharomyces pastorianus* extract and their mechanisms of action available. This study aimed to investigate the in vivo and in vitro hypoglycemic effect of *S. pastorianus* extract and to elucidate its molecular mechanisms. *S. pastorianus* extract was mainly composed of proteins followed by carbohydrates. In diabetic rats, oral administration of *S. pastorianus* extract significantly reduced the levels of plasma glucose and enhanced the activity of hepatic glucose-6-phosphatase dehydrogenase. Treatment with *S. pastorianus* extract increased the localization of type 4 glucose transporter (GLUT4), PTP, and insulin receptor at 3T3-L1 cell membranes and raised the levels of P38 MAPK, PI3K, and AKT in the cytosol. In agreement with these results, pretreatment of 3T3-L1 cells with inhibitors of PTP, PI3K, Akt/PKB, and p38 MAPK inhibited glucose uptake induced by application of *S. pastorianus* extract. Most importantly, a 54 kDa protein with hypoglycemic activity was identified and suggested as the major ingredient contributing to the hypoglycemic activity of *S. pastorianus* extract. In summary, these results clearly confirm the hypoglycemic activity of *S. pastorianus* extract and provide critical insights into the underlying molecular mechanisms.

## 1. Introduction

Hyperglycemia is the presence of excessively high plasma glucose levels due to reduced glucose uptake by cells and is a characteristic of diabetes [1]. Long-term complications caused by hyperglycemia include heart disease, stroke, diabetic retinopathy and resulting blindness, kidney failure, and poor blood flow to the extremities leading to amputations [2]. Glucose uptake is regulated by several mechanisms and cellular components, of which insulin plays the most prominent role. Insulin promotes the absorption of glucose mainly by adipose and skeletal muscle cells via movement of the type 4 glucose transporter (GLUT4) from the cytosol to the cell membrane. This translocation of GLUT4 depends on multiple steps in the PI3K/AKT and p38 MAPK signaling pathways [3].

Hyperglycemia occurs both in diabetic patients with insulin resistance and in patients with excess endogenous glucose production. Hepatic control of endogenous glucose homeostasis is achieved via the coordination of signaling pathways regulating glycogen synthesis, glycogenolysis, and gluconeogenesis. Glucose-6-phosphatase (G6Pase) and hexokinase play crucial roles in endogenous glucose production, and a reduction in the ratio of G6Pase to hexokinase is considered a good prognostic indicator for type 2 diabetes [4,5]. Additionally, interactions have been reported between diabetes and a deficiency in glucose-6-phosphate dehydrogenase (G6PD), the enzyme that catalyzes the first step of the pentose phosphate pathway. Moreover, G6PD deficiencies are highly correlated with the prevalence of diabetes [6].

Besides insulin therapy, diabetic hyperglycemia patients often receive oral hypoglycemic agents; however, there are concerns regarding many synthetic hypoglycemic drugs, which cause adverse reactions [7]. Moreover, naturally sourced biologically active compounds have the potential to treat various diseases and also play major roles in the drug development process. Compared with purely synthetic drugs, natural products are generally considered safer, cheaper, easier to obtain, and sometimes even more effective [8]. Much work has thus been done to identify natural products with hypoglycemic activity from multiple types of organisms. Compared with plants, the use of microorganisms to produce hypoglycemic substances enables easier access to raw materials and lower production costs. Previously, we determined the optimal cultural medium and conditions for the proliferation of *S. pastorianus* no. 54 isolated from soils of a winery and evaluated the hypoglycemic potential of *S. pastorianus* extracts in rat epididymal adipocyte, and differentiated 3T3-L1 adipocyte models [9,10]. However, identities of the biologically active ingredients in *S. pastorianus* extract and the mechanisms underlying its function are not completely clear.

The purpose of this study was to investigate the hypoglycemic activity of *S. pastorianus* extract in nicotinamide/streptozotocin-induced diabetic rats, and to elucidate whether its underlying mechanism operates via the regulation of hepatic carbohydrate metabolism enzymes. In addition, the activation of key signaling pathways involved in GLUT4 expression was examined using the differentiated 3T3-L1 adipocytes. Finally, the protein from *S. pastorianus* extract exhibiting hypoglycemic activity was identified and characterized.

## 2. Materials and Methods

**Scheme 1:**
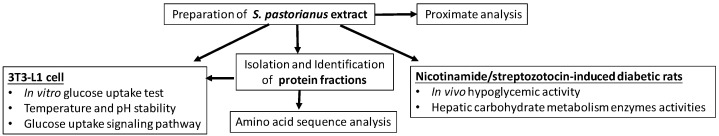
Flow chart of *S. pastorianus* extract preparation and analysis, protein isolation and identification, in vivo and in vitro hypoglycemic activity evaluation, and underlying mechanism elucidation.

The flow chart of whole study is shown as Scheme 1.

### 2.1. Cell Lines, Chemicals, and Reagents

The strain of *S. pastorianus* no. 54 was isolated by our research group, as described previously [10]. The 3T3-L1 cell line (BCRC CL-173) was purchased from Bioresource Collection and Research Center (Hsinchu, Taiwan). All chemicals, reagents, and inhibitors for glucose uptake tests were purchased from Sigma Chemical (St. Louis, MO, USA) unless otherwise stated. Culture media for *S. pastorianus* and 3T3-L1 adipocytes were purchased from Difco Laboratories (Detroit, MI, USA) and Gibco (New York, NY, USA), respectively. The rat insulin ELISA kit was purchased from Randox Laboratories, Ltd. (Crumlin, UK). The glucose detection kit was purchased from Audit Diagnostics (Cork, Ireland). Pre-stained SDS-PAGE standards were purchased from Pharmacia (Uppsala, Sweden). Acrylamide, *N*,*N*’-methylene-bis-acrylamide (Bis), *N*,*N*,*N*’,*N*’-tetra-methylethylene-diamine (TEMED), ammonium persulfate, tris (hydroxymethyl) aminomethane, 2-mercaptoethanol, sodium dodecyl sulfate, and a Bio-Rad DC protein assay kit were purchased from Bio-Rad (Hercules, CA, USA).

### 2.2. Preservation, Culture and Preparation of S. pastorianus Extract

Based on the method of Wu et al., *S. pastorianus* no. 54 was stored at −80 °C in malt extract broth (MEB) containing 50% sterile glycerol [10]. It was sub-cultured twice in MEB at 25 °C for 48 h, and the activated culture was inoculated into a 5 L-volume fermenter containing 3 L medium (pH 6.0) of diluted strawberry juice (with reducing sugar content of 1.74 g/L) plus 1% glucose and 0.1% yeast extract with initial cell density of 1 × 10^5^ CFU/mL and incubated at 20 °C, 150 rpm, 1.2 vvm for 4 days. After centrifugation (5000× *g*, 20 min), the precipitate was washed three times with deionized water and then freeze-dried. One gram of freeze-dried yeast powder was added to 20 mL of 0.1 N NH_4_OH aqueous solution and incubated at 30 °C in water bath with shaking for 2 h. After centrifugation (10,000× *g*, 15 min), the supernatant was collected and freeze-dried. The freeze-dried extract was subsequently used for composition analysis, protein isolation and hypoglycemic activity evaluation.

### 2.3. Proximate and Amino Acid Analysis of S. pastorianus Extract

The proximate compositions of extracts, including crude ash, crude fat, and crude protein, were determined according to the methods of AOAC (1990). The carbohydrate content was calculated by subtracting the contents of crude ash, fat, and protein from dry matter [11]. The extract (0.1 g) was added with HCl (10 mL, 6 M) in a vacuum vial and digested at 110 °C for 24 h to liberate amino acids from proteins. The preparation of trichloroacetic acid extract and the analysis of free amino acids was carried out in the same way as described in the previous study [12]. Quantitative amino acid analysis was conducted using a Beckman System 6300 amino acid analyzer (Beckman Instruments, Inc., Brea, CA, USA). Chromium content in extract was analyzed by atomic absorption spectroscopy) (Z-5300, Hitachi, Tokyo Japan), based on the method of Pourjavid et al. [13].

### 2.4. Temperature and pH Stability of the Hypoglycemic Activity of S. pastorianus Extract

The *S. pastorianus* extract solution (250 μg/mL) was heated at 35 °C, 45 °C, 55 °C, 65 °C, 75 °C, and 85 °C for 2 h, and the residual hypoglycemic activity was measured at 10 min interval. In a parallel experiment, the extract solution (250 μg/mL) was adjusted to various pH values (pH 2.0–9.0) and incubated at 25 °C for 2 h. The residual hypoglycemic activity of this solution was measured.

### 2.5. Hypoglycemic Activity Analysis by 3T3-L1 Cell Line

Based on the protocol of Student et al., the differentiation of 3T3-L1 preadipocytes was initiated with 1 μg/mL insulin, 1 μM dexamethasone, and 0.5 mM 3-iso-butyl-1-methylxanthine in DMEM supplemented with 10% fetal bovine serum (FBS) [14]. After incubation at 37 °C for 48 h, the culture medium was replaced with DMEM supplemented with 10% FBS and 1 μg/mL insulin and was incubated for an additional 48 h. Cells were then fed with DMEM containing 10% FBS every other day. The cells were fully differentiated into adipocytes at the end of day 10, as monitored by Oil Red-O staining [15]. The differentiated 3T3-L1 adipocytes in the microtiter plate were put to a state of fasting in DMEM without glucose at 37 °C for 1 h. After being washed with PBS, 200 μL of sample (250 μg/mL) and 200 μL of PBS containing 10 nM insulin and 2.5 g/L glucose were added. To investigate the influence of various inhibitors on glucose uptake, differentiated 3T3-L1 adipocytes were cultured in the presence of sodium orthovanadate (20 μΜ, PTP inhibitor), wortmannin (0.88–10 nM, PI3 kinase inhibitor), bisindolylmaleimide (0.88–10 nM, PKC inhibitor), Akt/PKB inhibitor (1.25–10 M), or SB203580 (10 μM, p38 MAPK inhibitor) for 20 min prior to extract treatment. The cultures were further incubated at 37 °C for 2 h. After centrifugation (400× *g*, 15 min), glucose content in the supernatant was determined by glucose detection kit following the supplier’s instruction. Increased glucose uptake percentage (%) = ((glucose uptake of cells with sample−glucose uptake of control)/glucose uptake of control) × 100%.

### 2.6. Animals and Experimental Design

Male Sprague-Dawley rats, 8 weeks old, were obtained from BioLASCO Taiwan Co., Ltd. (Taipei, Taiwan). On arrival, rats were housed in stainless steel cages with free access to diet and water for 1 week before treatment. Rats were kept in a humidity (30–70%), light (12-h light/dark cycle), and temperature (23 ± 2 °C)-controlled environment. The study was conducted in accordance with the Declaration of Helsinki, and the protocol was approved by the National Taiwan Ocean University Institutional Animal Care and Use Committee.

The rats were randomly divided into 3 groups (n = 8): normal control, diabetic control, diabetic rats fed with *S. pastorianus* extract (45 mg/kg b.w./day). For induction of diabetes, the rats were subcutaneously injected with nicotinamide (230 mg/kg b.w.) and streptozotocin (65 mg/kg b.w.) with a 15-min interval [16]. After 1 week, an oral glucose tolerance test (OGTT; 1.5 g glucose per kg body weight of rat) was performed to confirm the successful induction of diabetes, and the rats were then treated with *S. pastorianus* extract by gavage daily for 6 weeks. After fasting for 12 h, the blood samples were collected for the determination of plasma level of glucose and insulin. OGTT was performed again at the 6th week after diabetes induction, and the samples of liver were harvested after sacrifice. The levels of hexokinase, G6Pase, and G6PD in the liver were measured as described previously [17,18,19,20]. Briefly, the liver samples were homogenized in N-acetyl-cysteine buffer (liver/buffer = 1/10; 4 mM N-acetyl-cysteine, 4 mM EDTA, 0.15 M KCl, 4 mM MgSO_4_, pH 7.0) and centrifuged (15,000× *g* at 4 °C for 40 min). The supernatants (0.1 mL) were mixed with glycylglycine buffer (0.6 mL, 0.25 M, pH 7.5), MgSO_4_ (0.3 mL, 0.75 M), NADP^+^ (0.2 mL, 7.5 mM), glucose (0.4 mL, 0.75 M), KCl (0.3 mL, 1.0 M), G6PD (10 μL, 7 unit/mL), H_2_O (0.79 mL), and ATP (0.2 mL, 0.75 M). Optical density at 340 nm of the mixture was measured by an ELISA reader (μ Quant, BIO-TEK, Winooski, VT, USA) every 30 s for 5 min to determine the activity of hexokinase. To determine the activity of G6Pase, the supernatants (0.2 mL) were mixed with Tris-HCl buffer (2 mL, 0.05 M, pH 7.5), MgCl_2_ (0.1 mL, 0.2 M), G6P (0.1 mL, 0.025 M), NADP^+^ (0.1 mL, 7.5 mM), and H_2_O (0.6 mL) for 5 s. Optical density at 340 nm of the mixture was measured every 30 s for 5 min. On the other hand, the supernatants (0.2 mL) were mixed with Tris-maleate buffer (0.6 mL, 0.05 M, pH 7.5) and glucose-6-phosphate (0.2 mL, 0.1 M) and incubated in a 37 °C water bath for 40 min followed by addition of trichloroacetic acid (4 mL, 5%). After centrifugation (3000× *g* for 30 min), the supernatants (1 mL) were mixed with H_2_SO_4_ (0.5 mL, 5 N), ammonium molybdate tetrahydrate (0.5 mL, 2.5%), ascorbic acid (0.1 mL, 10%), and H_2_O (4 mL). Optical density at 660 nm of the mixture was measured every 15 s for 3 min to determine the activity of G6PD.

### 2.7. Detection of GLUT4, PTP, and Insulin Receptor Expression on Cell Membrane and P38 MAPK, PI3K, and AKT/PKB Levels in Cytosol of 3T3-L1 Cells

After treatment of the differentiated 3T3-L1 cell culture with glucose (2.5g/L; blank), insulin (10 nM; control), or *S. pastorianus* extract (250 μg/mL; sample) for 2 h, the cells were collected, disrupted in a cold lysis buffer (Tris, 50 mM; EDTA, 10 mM; Triton X-100, 1% v/v; phenylmethylsulphonyl fluoride, 1 mM; pepstatin A, 0.05 mM and leupeptin, 0.2 mM) and then centrifuged (10,000× *g*, 30 min). The protein content in the supernatant was measured with the protein assay kit (Bio-Rad Laboratories) using bovine serum albumin (BSA) as the standard. After being boiled for 10 min in a ratio of 1:1 with gel loading buffer (Tris, 50 mM; SDS, 10% *w/v*; glycerol, 10% *v/v*; 2-mercaptoethanol 10% *v/v* and bromphenol blue, 2 mg·mL^–1^), cellular proteins in supernatant (20 μg in total) were separated by 12% SDS-PAGE and transferred to a polyvinylidene fluoride membrane using a Labconco semi-dry blotter (Labconco, Kansas, MO, USA). The blot was incubated in TBST buffer (10 mM Tris, 75 mM NaCl, 1 mM EDTA, pH 7.4, containing 0.1% Tween 20^®^) containing 5% BSA for 1 h and then primed with rabbit anti-murine GLUT4 antibody (Santa Cruz Biotechnology, Inc., Santa Cruz, CA, USA) for 1 h. After washing with TBST buffer 4 times, the blot was incubated with peroxidase conjugated goat anti rabbit-IgG antibody for 1 h. The bands of the GLUT4 blot were visualized using enhanced chemiluminescence system and quantified using a laser densitometer with Quantity-One software (Amersham Biosciences, Little Chalfont, Buckinghamshire, UK).

For immunofluorescent staining, cell pellets were suspended in staining buffer (2% FBS and 0.1% sodium azide in PBS) containing anti-GLUT4, anti-PTP, or anti-insulin receptor primary antibodies for 30 min-incubation, washed with staining buffer 3 times, and followed by incubation in the presence of FITC-conjugated secondary antibodies for 30 min. On the other hand, parts of cell were fixed with an equivalent volume of Phosflow Fix Buffer I for 10 min at 37 °C. Fixed cells were pelleted and re-suspended in Phosflow perm/wash Buffer I and permeabilized for 30 min on ice. Cells were washed and stained with anti-PI3K, anti-PKC, anti-Akt, or anti-P38 MAPK antibodies (Abgent Inc., San Diego, CA, USA) followed by FITC-conjugated secondary antibodies (Jackson Immuno Research Lab Inc., West Grove, PA, USA.) The expression levels of the above molecules were determined by a BD FACSCantoTM flow cytometer (BD Biosciences, San Jose, CA, USA).

### 2.8. Isolation and Identification of Hypoglycemic Protein Contained in S. pastorianus Extract

One gram of lyophilized extract powder was dissolved in 5 mL phosphate buffered saline (PBS, pH 7.0) and subjected to diethylaminoethyl (DEAE) cellulose anion exchange resin column (2.5 × 30 cm, Bio-Rad Laboratories). The column was washed with PBS (pH 7.0) and eluted with a linear gradient of 0 to 0.1 N HCl in PBS at a flow rate of 0.1. Ten mL per tube were collected and monitored by absorption at 280 nm. The unabsorbed protein fraction (DC1) was concentrated by Amicon ultrafiltration and further loaded onto a DOWEX 50WX8-200 cation exchange resin column (2.5 × 30 cm, Bio-Rad Laboratories) using Na_3_PO_4_-HCl buffer (pH 7.0) as the rinsing solution and eluted with Na_3_PO_4_ (0–0.2 M) at a flow rate of 1 mL/min. Ten milliliters per tube were collected and monitored by absorption at 280 nm. Four peak fractions of the un-absorbed peak and absorbed peak from the DEAE cellulose anion exchange column and DOWEX 50WX8-200 cation exchange resin column were pooled for hypoglycemic activity analysis and sodium dodecyl sulfate-polyacrylamide gel electrophoresis (SDS-PAGE) analysis

Ten microliters of the above peak fractions (2 mg protein/mL) was added with Tris-HCl (0.5 M, pH 6.8) and heated at 95 °C water bath for 5 min. The samples were subjected to SDS-PAGE analysis (acrylamide concentration: stacking gel: 5%; resolving gel: 15%), and then the gel was stained with Coomassie brilliant blue G-250 according to the protocol of Neuhoff et al. [21]. The protein bands in the gel were cut out and subjected for hypoglycemic activity measurement and amino acid sequence analysis (Genomics Inc., New Taipei, Taiwan).

### 2.9. Statistical Analysis

Data were expressed as the mean ± standard deviation (SD) for each treatment group. Independent sample *t*-test was used to assess the statistical difference between each group. *p*-values of less than 0.05 were defined as statistically significant.

## 3. Results

### 3.1. The Composition of S. pastorianus Extract and the pH and Temperature Stability of Its Hypoglycemic Activity

**Figure 1:**
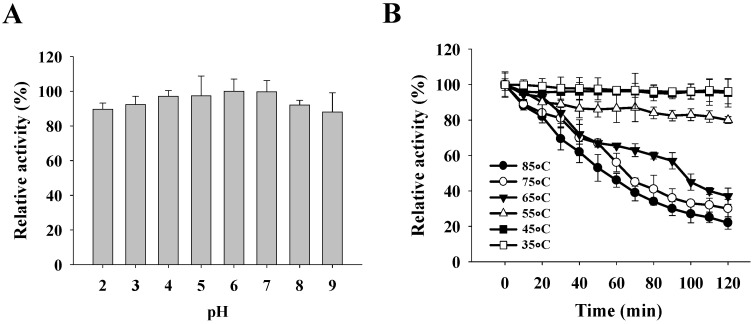
pH and thermal stabilities of the hypoglycemic activity of *S. pastorianus* extract using differentiated 3T3-L1 cell assay model. (**A**) Relative activity of *S. pastorianus* extract on facilitating glucose uptake after heat-treatment at various temperatures for 0–120 min. (**B**) Relative activity of *S. pastorianus* extract on facilitating glucose uptake after exposure to solutions with different pH for 2 h.

*S. pastorianus* extract was mainly composed of proteins (62.45% ± 1.14%) followed by carbohydrates (28.11% ± 0.05%), with the major amino acids present being glutamic acid, aspartic acid, lysine, alanine, glycine, and serine (Table 1 and Table 2). In our previous study, *S. pastorianus* extract treatment significantly increased glucose uptake by the differentiated 3T3-L1 adipocytes [9]. Herein, we further evaluated the impact of pH and temperature on *S. pastorianus* extract-induced glucose uptake in 3T3-L1 adipocytes. As shown in Figure 1A, *S. pastorianus* was quite stable and maintained 95–100% relative hypoglycemic activity over a pH range of 4.0 to 7.0. Even when incubated at extreme acidic pH values of 2.0 and 3.0 for 2 h, *S. pastorianus* extract retained about 85% of the activity. The relative hypoglycemic activity of the *S. pastorianus* extract remained >95% when incubated at 35–45 °C for 2 h. However, when the temperature was raised to 45 °C or higher, the relative activity of the extract gradually decreased (Figure 1B).

### 3.2. Oral Administration with S. pastorianus Extract Reduces Plasma Glucose Levels and Modulated the Activity of Hepatic Carbohydrate Metabolism Enzymes in Diabetic Rats

**Figure 2:**
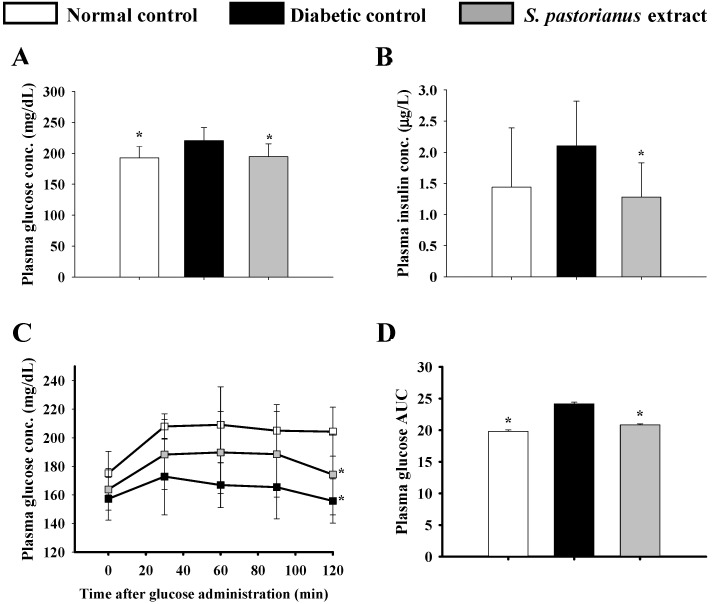
The level of plasma glucose in rats treated with *S. pastorianus* extract. The rats were injected with nicotinamide and streptozotocin for the induction of diabetes and fed with *S. pastorianus* extract for 6 weeks as described in Materials and Methods. After fasting for 12 h, the blood samples were collected for the determination of fasting plasma level of (**A**) glucose and (**B**) insulin. After oral administration of glucose, the blood samples were collected at 0, 30, 60, and 120 min for the determination of (**C**) plasma glucose and (**D**) the area under the curve (AUC) of plasma glucose concentration. Results are expressed as mean ± SD for each group of rats (*n* = 8). * *p* < 0.05 compared with diabetic control.

To investigate the in vivo hypoglycemic activity of *S. pastorianus* extract, a rat model of nicotinamide/streptozotocin-induced diabetes was employed. After fasting for 12 h, the plasma levels of glucose and insulin of diabetic rats were measured and found to be higher than that of normal rats. However, treatment with *S. pastorianus* extract significantly reduced plasma glucose and insulin levels (Figure 2A,B). Additionally, in vivo hypoglycemic activity caused by *S. pastorianus* extract application was confirmed using OGTT. After oral glucose administration, plasma glucose levels were markedly elevated in diabetic rats compared with those in normal control rats, indicating successful induction of diabetes (Figure 2C). By contrast, plasma glucose levels were significantly reduced in rats treated with *S. pastorianus* extract compared with those in diabetic rats (Figure 2C). Moreover, the plasma glucose area under the curve (AUC) in diabetic rats was larger than that of normal control rats, whereas treatment with *S. pastorianus* extract greatly decreased glucose AUC (Figure 2D).

**Figure 3:**
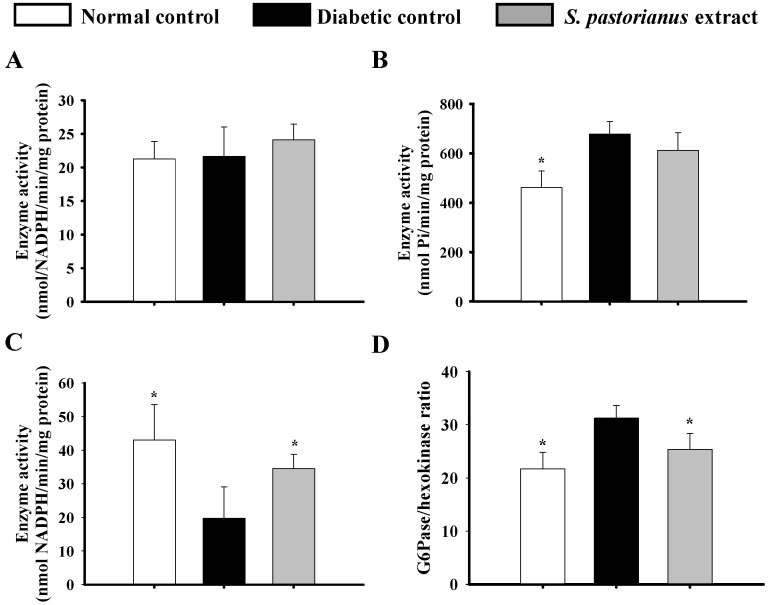
The carbohydrate metabolism enzymes activities in liver of diabetic rats treated with *S. pastorianus* extract. The rats were injected with nicotinamide and streptozotocin for the induction of diabetes and fed with *S. pastorianus* extract for 6 weeks as described in Materials and Methods. After sacrifice, the liver samples were harvested for the detection of (**A**) hexokinase, (**B**) glucose-6-phosphatase (G6Pase), (**C**) glucose-6-phosphate dehydrogenase (G6PD), and the ratio of G6Pase/hexokinase (**D**) activities. Results are expressed as mean ± SD for each group of rats (*n* = 8). * *p* < 0.05 compared with diabetic control.

These results clearly demonstrated the hypoglycemic effect of *S. pastorianus* extract in diabetic rats. Accordingly, we further examined the influence of *S. pastorianus* extract on the activities of hepatic carbohydrate metabolism enzymes. Hexokinase activity levels were comparable between each group (Figure 3A), whereas the activity of G6Pase and G6PD was up-regulated and down-regulated in diabetic control rats, respectively (Figure 3B,C). Compared with diabetic control rats, G6PD activity levels were significantly increased, and the G6Pase/hexokinase ratio was reduced in rats treated with *S. pastorianus* extract (Figure 3C,D). These findings suggest that modulation of hepatic carbohydrate metabolism enzyme is one of the major mechanisms underlying the hypoglycemic effect of *S. pastorianus* extract.

### 3.3. The Role of GLUT4 Expression and P38 MAPK, PI3K, and AKT/PKB Activation in S. pastorianus Extract-Induced Glucose Uptake in 3T3-L1 Cells

**Figure 4:**
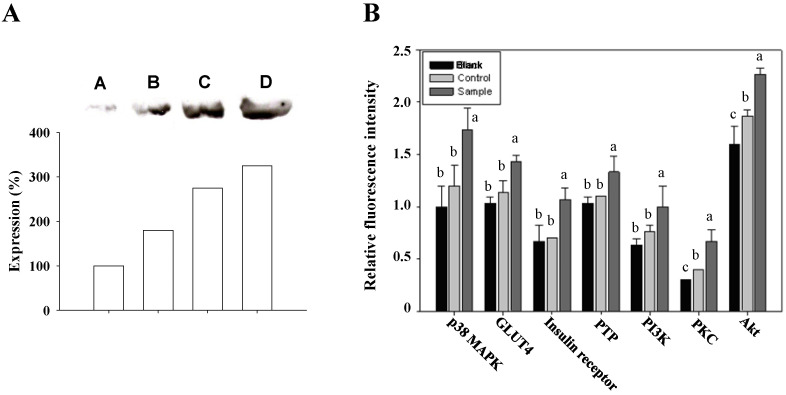
Effect of *S. pastorianus* extract on the GLUT 4, PTP, insulin receptor, and signal transduction protein expression in 3T3-L1 cells. (**A**) GLUT4 Western blot of cell membrane preparation of treated 3T3-L1 cells. Histogram representing the densitometric analysis. A: control; B: *S. pastorianus* extract; C: Insulin; D: Insulin + *S. pastorianus* extract. GLUT4 expression percentage was relative to control, where control was considered as 100% expression. (**B**) Effect of *S. pastorianus* extract on the expression of PTP and insulin receptor in cell membrane and some signal transduction proteins in the cytosol of differentiated 3T3-L1 cells evaluated by flow cytometry analysis as described in Materials and Methods. Blank: cells treated with glucose alone; Control: cells treated with glucose and insulin; Sample: cells treated with glucose, insulin, and *S. pastorianus* extract. Data represent means ± SD (n = 3). Different letters (a, b, c) on the bars are significantly different in each group (*p* < 0.05).

To clarify the mechanism underlying the hypoglycemic effect of *S. pastorianus* extract, the expression level of GLUT4 at 3T3-L1 cell membranes was determined by Western blotting. Compared with untreated cells, *S. pastorianus* extract treatment markedly increased GLUT4 expression (Figure 4A). Notably, a synergistic effect between *S. pastorianus* extract and insulin on the promotion of GLUT4 expression was observed in 3T3-L1 cells (Figure 4A). We also examined the levels of membrane-localized GLUT4, PTP, and insulin receptors and cytoplasmic P38 MAPK, PI3K, PKC, and AKT/PKB in 3T3-L1 cells using immunofluorescence staining to explore the activation of related signaling pathways. Compared with untreated cells, the average fluorescence intensity of the above targets was significantly increased, indicating that all these receptors and signal transduction proteins are involved in the regulation of cell hypoglycemia by *S. pastorianus* extract (Figure 4B).

**Figure 5:**
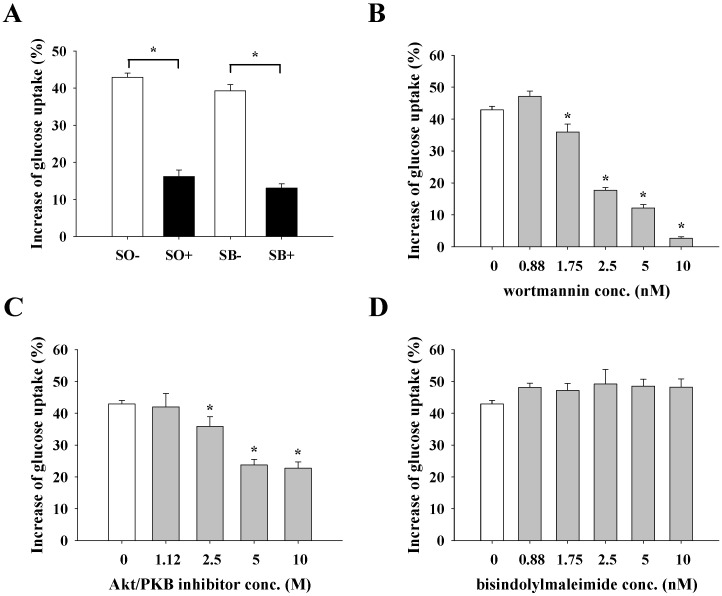
Impact of PTP and signal transduction protein inhibitors on hypoglycemic activity of *S. pastorianus* extract in 3T3-L1 cells. Pretreatment of 3T3-L1 cells with (**A**) sodium orthovanadate (SO; PTP inhibitor) and SB20358 (SB; p38 MAPK inhibitor), (**B**) wortmannin (PI3K inhibitor), (**C**) AKT/PKB inhibitor, and (**D**) bisindolylmaleimide (PKC inhibitor) for 20 min followed by *S. pastorianus* extract treatment for 2 h. The level of glucose in medium was determined to obtain the increased rate of glucose uptake compared to that before treatment. * *p* < 0.05 compared to cells treated without inhibitors.

To verify the role of signal transduction proteins in glucose uptake induced by *S. pastorianus* extract, 3T3-L1 cells were treated with inhibitors of PTP, PI3 kinase, PKC, and Akt/PKB before *S. pastorianus* extract application. Both sodium orthovanadate (20 μM) and SB203580 (10 μM) pretreatment significantly inhibited the cell’s glucose uptake induced by *S. pastorianus* extract (Figure 5A). Additionally, pretreatment with wortmannin (0.88–10 nM) and an Akt/PKB inhibitor (1.25–10 M) reduced glucose uptake in a concentration-dependent manner (Figure 5B,C). Interestingly, pretreatment with bisindolylmaleimide (0.88–10 nM) did not significantly affect glucose uptake (Figure 5D). Taken together, these results indicate that the activation of PTP, PI3K, AKT/PKB, and p38 MAPK, but not PKC, was essential for glucose uptake induced by *S. pastorianus* extract.

### 3.4. Harvest of a Hypoglycemic Protein Fraction from S. pastorianus Extract Using Ion Exchange Column Chromatography

**Figure 6:**
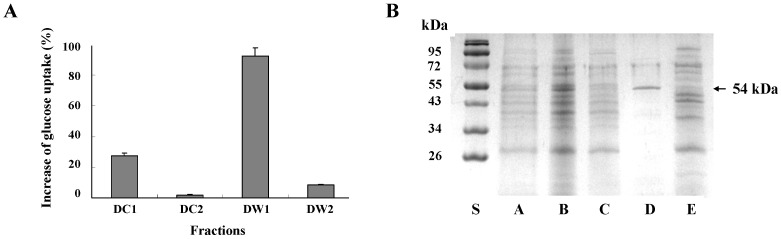
Effect of protein fractions harvested from *S. pastorianus* extract on glucose uptake of 3T3-L1 cells. (**A**) Increased glucose uptake percentage in 3T3-L1 cells treated with various fractions. (**B**) SDS-PAGE of *S. pastorianus* extract and fractions isolated from the DEAE cellulose column followed by DOWEX 50WX8-200 column chromatographs of *S. pastorianus* extract. S: standard markers; A: *S. pastorianus* extract; B: DC1; C: DC2; D: DW1; E: DW2.

*S. pastorianus* extract was separated using DEAE cellulose column chromatography, and two protein fractions, including DC1 (unabsorbed fraction) and DC2 (absorbed-and-eluted fraction), were collected. The DC1 fraction was further passed through a DOWEX 50WX8-200 cation exchange resin column, and fractions DW1 (unabsorbed fraction) and DW2 (absorbed-and-eluted fraction) were collected. As shown in Figure 6A, the unabsorbed fractions DC1 and DW1 had hypoglycemic activity, with DW1 promoting higher levels of glucose uptake. After SDS-PAGE analysis, a protein with a molecular weight of 54 kDa (DW1) was obtained (Figure 6B), which was further eluted from the gel and proved to have good hypoglycemic activity and did not contain chromium (data not shown). Amino acid sequence analysis revealed that this protein consisted of 416 amino acids (Table 3). The DW1 fraction, which also contained the single protein of 54 kDa, had the strongest efficacy to promote glucose uptake among the four tested fractions, and this 54 Da protein had been proved to increase glucose uptake in differentiated 3T3-L1 adipocytes. Accordingly, we suggest that this 54 kDa protein is the main component for the hypoglycemic effect of *S. pastorianus* extract.

## 4. Discussion

Although the hypoglycemic potential of brewer’s yeast extract has been reported, there is limited information pertaining to the hypoglycemic ingredients of *S. pastorianus* extract and their mechanisms of action available. In this study, we identified a hypoglycemic protein and the molecular mechanism of *S. pastorianus* extract in vivo and in vitro. This assertion is substantiated by several lines of evidence. First, daily treatment with *S. pastorianus* extract for 6 weeks significantly down-regulated the levels and AUC of plasma glucose in diabetic rats. The activity of hepatic G6PD was up-regulated and the ratio of G6Pase/hexokinase down-regulated in rats treated with *S. pastorianus* extract. Second, *S. pastorianus* extract treatment increased GLUT4 expression at the cell membrane, and the activation of PTP, PI3K, AKT/PKB, and p38 MAPK was essential for *S. pastorianus* extract-induced glucose uptake. Most importantly, a 54 kDa protein from *S. pastorianus* extract was identified, which significantly increased glucose uptake by 3T3-L1 cells. Our data clearly substantiate the hypoglycemic activity of *S. pastorianus* extract and provide critical insights to the underlying mechanisms of action.

So far, only a few studies have demonstrated the hypoglycemic potential of yeast, and there is still controversy over the mechanisms responsible. Mazza et al. detected a significant reduction in serum glucose levels in subjects orally treated with red yeast rice daily, with Monacolin K and coenzyme Q10 considered the major ingredients contributing to these pharmacological activities [22]. Beneficial effects of chromium-rich yeast on glucose tolerance in elderly subjects have also been reported [23,24]. Additionally, oral administration of spray-dried high-chromium yeast significantly delayed the onset of hyperglycemia in type 2 diabetic KK-AY mice and significantly reduced fasting blood glucose. The suggested potential mechanism underlying such effects of high-chromium yeast was the improvement of glucose metabolism by increasing the level of insulin receptors [25]. Moreover, baker’s yeast glucan (BYG) has been reported to be an anti-diabetic agent. Oral administration of BYG decreased blood and hepatic glucose levels and the prevalence of lipid disorders in ob/ob mice. BYG appears to up-regulate p-AKT and p-AMPK and down-regulate genes responsible for gluconeogenesis, explaining its function in mice [26].

**Figure 7:**
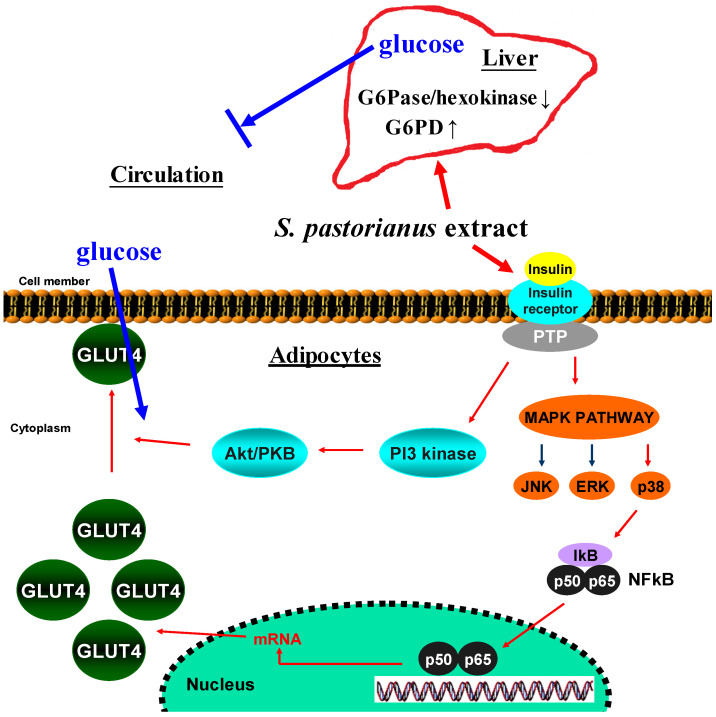
The proposed mechanism of the *S. pastorianus* extract-induced hypoglycemic effect targeting adipocytes and hepatocytes. The *S. pastorianus* extract could increase the sensitivity of insulin receptor and activate the membrane protein of phosphotyrosine phosphatase (PTP), which then activated PI3 kinase. The Akt/PKB kinase was activated by phosphoinositide-dependent kinase, which is a downstream signal of PI3 kinase. After the cascade of activation, the production of GLUT4 was up-regulated in cytosol and moved to the cell membrane for enhancement of glucose uptake. Simultaneously, S. pastorianus extract also elicited the p38 MAPK, which finally activated GLUT4. In addition, *S. pastorianus* extract augmented the activity of hepatic G6PD and reduced the ratio of G6Pase/hexokinase ratio for prevention against endogenous glucose overproduction.

Previously, we determined the optimal culture medium and conditions for the proliferation of *S. pastorianus* no. 54. The hypoglycemic activity of *S. pastorianus* no. 54 was first explored in 3T3-L1 adipocytes, rat epididymal adipocytes, and diabetic mice [9,10]. In this study, we isolated and identified a 54 kDa protein without chromium as the major active ingredient responsible for the hypoglycemic activity of *S. pastorianus* extract. We also examined the pH and temperature stability of *S. pastorianus* extract-induced hypoglycemia. Our results also indicate that *S. pastorianus* extract can enhance insulin receptor sensitivity and PTP activation, which then activates PI3K followed by a phosphoinositide-dependent kinase and Akt/PKB kinase. After this activation cascade occurred, increased levels of GLUT4 were produced in the cytosol and then moved to the cell membrane for glucose uptake (Figure 7). Simultaneously, *S. pastorianus* extract elicited the activation of p38 MAPK, which plays an important role in GLUT4 activation (Figure 7) [27]. Notably, the expression of PKC was raised in 3T3-L1 cells treated with *S. pastorianus* extract (Figure 3B). However, pretreatment of 3T3-L1 cells with bisindolylmaleimide, an inhibitor for the isoforms of PKCα, PKCβI, PKCβII, and PKCγ [28], showed limited impact on *S. pastorianus* extract-induced glucose uptake. Further studies are merited to comprehensively verify whether and which PKC isoforms play an essential role in *S. pastorianus* extract-induced glucose uptake. In addition, *S. pastorianus* extract also modulated hepatic G6PD activity and the G6Pase/hexokinase ratio, which are critical for carbohydrate metabolism regulation and the prevention of endogenous glucose overproduction (Figure 7) [4,5,6].

In the study of Chao et al. [29], hypoinsulinemia was observed one week after the injection of nicotinamide/streptozotocin for type 2 diabetic rats. However, there was no follow-up study to monitor the level of insulin for a longer time. In this study, the level of insulin was detected 7 weeks after the injection of nicotinamide/streptozotocin. Consistent with the results of previous study employing the same model [16], the level of insulin of normal rats and diabetic rats was comparable. Therefore, it is suggested that the employed diabetic model could not completely damage pancreatic beta cells, and the function of beta cells would be recovered as time goes by. Moreover, the employed model and treatment did not affect body weight, adiposity, and food intake (data not shown). These results are consistent with that of previous study employing the same model [16].

## 5. Conclusions

In conclusion, our data clearly demonstrated the in vivo and in vitro hypoglycemic activity of *S. pastorianus* extract. This activity was closely associated with enhanced GLUT4 expression, insulin signal transduction pathway activation, and the modulation of hepatic carbohydrate metabolism enzymes. Because of the advantages of yeast natural products and their hypoglycemic activity, *S. pastorianus* extract is considered a promising candidate for development as a functional food or medicine for hyperglycemia management.

## Figures and Tables

**Table 1:** Proximate compositions of *S. pastorianus* extract.

Composition	(%)
Moisture	1.81 ± 0.05
Carbohydrate	28.11 ± 0.98
Crude protein	62.45 ± 1.14
Crude lipid	0.70 ± 0.04
Ash	6.93 ± 0.19

Each value represents the mean ± S.D. (*n* = 3).

**Table 2:** Amino acid compositions of *S. pastorianus* extract.

Amino Acids	Protein Hydrolysate		Free Amino Acid	
	μmole/mL	g/100 g	μmole/mL	g/100 g
Glycine	1.26	2.37	0.08	0.14
Alanine	1.08	2.39	0.46	1.01
Valine	0.57	1.66	0.10	0.29
Leucine	0.60	1.97	0.07	0.23
Isoleucine	0.06	0.19	0.06	0.19
Methionine	0.12	0.44	0.00	0.00
Phenylalanine	0.30	1.25	0.04	0.17
Tyrosine	0.22	1.01	0.03	0.16
Tryptophan	0.01	0.05	0.01	0.05
Serine	0.90	2.35	0.09	0.23
Proline	0.71	2.04	0.37	1.05
Threonine	0.46	1.38	0.04	0.12
Cysteine	0.28	1.66	0.04	0.23
Asparagine	0.00	0.00	0.02	0.05
Glutamine	0.00	0.00	0.99	3.61
Lysine	0.77	2.83	0.77	2.80
Histidine	0.22	0.86	0.05	0.19
Arginine	0.36	1.54	0.04	0.16
Aspartic acid	1.09	3.62	0.13	0.42
Glutamic acid	2.80	10.29	0.74	2.73
Total	11.81	37.90	4.13	13.83

**Table 3:** The amino acid sequence of 54 kDa protein from *S. pastorianus* extract.

1 MSLSSKLSVQ DLDLKDKRVF IRVDFNVPLD GKKITSNQRI VAALPTIKYV 51 LEHHPRYVVL ASHLGRPNGE RNEKYSLAPV AKELQSLLGK DVTFLNDCVG 101 PEVEAAVKAS APGSVILLEN LRYHIEEEGS RKVDGQKVKA SKEDVQKFRH 151 ELSSLADVYI NDAFGTAHRA HSSMVGFDLP QRAAGFLLEK ELKYFGKALE 201 NPTRPFLAIL GGAKVADKIQ LIDNLLDKVD SIIIGGGMAF TFKKVLENTE 251 IGDSIFDKAG AEIVPKLMEK AKAKGVEVVL PVDFIIADAF SADANTKTVT 301 DKEGIPAGWQ GLDNGPESRK LFAATVAKAK TIVWNGPPGV FEFEKFAAGT 351 KALLDEVVKS SAAGNTVIIG GGDTATVAKK YGVTDKISHV STGGGASLEL 401 LEGKELPGVA FLSEKK

## Data Availability

The data presented in this study are available on request from the corresponding author.
